# Assessing Gender Differences in Neuropathic Pain Management: Findings from a Real-Life Clinical Cross-Sectional Observational Study

**DOI:** 10.3390/jcm13195682

**Published:** 2024-09-24

**Authors:** Gianmarco Marcianò, Antonio Siniscalchi, Gianfranco Di Gennaro, Vincenzo Rania, Cristina Vocca, Caterina Palleria, Luca Catarisano, Lucia Muraca, Rita Citraro, Maurizio Evangelista, Giovambattista De Sarro, Bruno D’Agostino, Diana Marisol Abrego-Guandique, Erika Cione, Bart Morlion, Luca Gallelli

**Affiliations:** 1 Operative Unit of Clinical Pharmacology and Pharmacovigilance, Renato Dulbecco University Hospital, Viale Europa, 88100 Catanzaro, Italy; gianmarco.marciano3@gmail.com (G.M.); raniavincenzo1@gmail.com (V.R.); cristina_vocca@live.it (C.V.); palleria@unicz.it (C.P.); lucacatarisano@gmail.com (L.C.); citraro@unicz.it (R.C.); desarro@unicz.it (G.D.S.); 2 Department of Neurology and Stroke Unit, Annunziata Hospital of Cosenza, Via Della Repubblica, 87100 Cosenza, Italy; anto.siniscalchi@libero.it; 3 Department of Health Science, Magna Graecia University, Viale Europa, 88100 Catanzaro, Italy; gianfranco.digennaro@unicz.it; 4 Research Center FAS@UMG, Department of Health Science, Magna Graecia University, Viale Europa, 88100 Catanzaro, Italy; 5 Department of Primary Care, ASP Catanzaro, 88100 Catanzaro, Italy; lalumuraca@gmail.com; 6 Department of Anesthesia, Resuscitation and Pain Therapy, Sacred Heart Catholic University, 00100 Rome, Italy; maurizio.evangelista@unicatt.it; 7 Department of Environmental Biological and Pharmaceutical Sciences and Technologies, University of Campania “Luigi Vanvitelli”, Viale Abramo Lincoln, 5, 81100 Caserta, Italy; bruno.dagostino@unicampania.it; 8 Department of Pharmacy, Health and Nutritional Sciences, University of Calabria, 87036 Rende, Italy; dianamarisol.abregoguandique@studenti.unicz.it (D.M.A.-G.); erika.cione@unical.it (E.C.); 9 The Leuven Centre for Algology and Pain Management, University Hospitals Leuven, 3000 Leuven, Belgium; bart.morlion@uzleuven.be; 10 Department of Cardiovascular Sciences, Section Anaesthesiology and Algology, KU Leuven—University of Leuven, 3000 Leuven, Belgium

**Keywords:** neuropathic pain, gender, treatment, efficacy, safety

## Abstract

**Introduction:** Neuropathic pain is defined as pain induced by a lesion or disease of the somatosensory nervous system. Pharmacological and non-pharmacological treatments are frequently employed. In the current clinical investigation, we assessed the effects of sex on the safety and effectiveness of medications used to treat neuropathic pain. **Methods:** We conducted a prospective analysis between 1 February 2021 and 20 April 2024, involving patients with neuropathic pain referred to the Ambulatory of Pain Medicine of “Renato Dulbecco” University Hospital in Catanzaro (Calabria, Italy). Patients over 18 years old with signs of neuropathic pain (Douleur Neuropathique en 4 questionnaire ≥ 4) were included. Exclusion criteria comprised patients with Alzheimer’s disease; patients with nociplastic or nociceptive pain; and patients with neoplasms. Patients with fewer than two accesses to ambulatory care were excluded, as were those who did not sign the informed consent. Clinical data were collected from each enrolled patient and subsequently analyzed, considering clinical outcomes. Sex and gender differences in efficacy were estimated using multivariate linear modeling and propensity-score matching. **Results:** During the study, 531 patients were screened, and 174 were enrolled (33.5%, mean age 61.5 ± 13.1; 64 males and 110 females, mean age 60.6 ± 13.4 and 61.96 ± 13.0) in accordance with the inclusion and exclusion criteria. Only minor differences in treatment prescription were observed based on age, body mass index, and comorbidities. Smoking, sex, educational level, and body mass index did not induce a significant change in pain perception. Males required slightly higher, though not significantly, doses of drugs for pain control than females. The treatment was not significantly more effective for females than for males. Females did not exhibit a significantly lower number of adverse drug reactions compared to males. **Conclusions:** The current study found that there are no appreciable differences between the sexes when it comes to the treatment of neuropathic pain.

## 1. Introduction

Neuropathic pain is a chronic condition caused by several clinical manifestations), accounting for 15–25% of chronic pain [1,2]. The International Association for the Study of Pain (IASP) defines neuropathic pain as pain caused by a lesion or disease of the somatosensory nervous system [3]. The pain is described as shooting, electrical-like, lancinating, and often irradiating according to the neuroanatomical nerve distribution (e.g., dermatomal, radicular) [3], which is relevant for the differentiation between neuropathic pain and nociplastic or nociceptive pain. Nociceptive pain is defined as arising from actual or threatened damage to non-neural tissue and is due to the activation of nociceptors [3]. Nociplastic pain is defined as altered nociception despite no clear evidence of actual or threatened tissue damage causing the activation of peripheral nociceptors or evidence for disease or lesion of the somatosensory system.

In a recent review of international guidelines and recommendations for the pharmacological treatment of neuropathic pain [4], we described the different therapeutic options. First-line drugs are antidepressants, including tricyclic antidepressants (TCA, e.g., amitriptyline) and serotonin noradrenaline reuptake inhibitors (SNRI: duloxetine and venlafaxine), but also antiepileptics (α2δ calcium channel unit blockers, pregabalin, and gabapentin). Some other substances, such as lidocaine patches, capsaicin 8% patches, and subcutaneous injections of botulinum toxin type A, have a weak recommendation and are only indicated for peripheral neuropathic pain [4]. Opioids are generally not recommended for the management of chronic non-cancer pain due to the long-term risks of side effects. However, some authors have suggested that the weak-opioid tramadol may be efficacious in the treatment of neuropathic pain [5].

ADRs are a serious concern for physicians managing subjects with pain (i.e., drowsiness and vertigo for antidepressants and antiepileptics, and dependence and stypsis for opioids), and these reduce the patients’ compliance [6,7,8]. In order to lower the ADRs onset rate (considering also its relationship with the prescribed dose and prolonged therapy), a non-pharmacological treatment may offer a safer therapeutic opportunity. Nutraceuticals are frequently used in individuals with pain, e.g., alpha-lipoic acid [9,10], acetyl-L-carnitine [11], and palmitoylethanolamide (PEA) [12,13]. Moreover, non-invasive techniques such as high intensity low-frequency pulsed magnetic fields (diamagnetic therapy) could represent an add-on treatment [14,15,16] due to their anti-inflammatory and anti-oedema effects [17,18].

According to the World Health Organization (WHO), gender refers to the characteristics of women, men, girls, and boys that are socially constructed, including norms, behaviors, and roles associated with being a woman, man, girl, or boy, as well as relationships with each other. Gender is related to many variables, including ethnicity, socioeconomic status, disability, age, geographic location, and sexuality. Conversely, sex refers to the different biological and physiological characteristics of the person, including karyotype, hormones, and reproductive organs [19].

Both males and females differ in their response to pain [20]. Indeed, females generally report higher pain sensitivity and intensity than males and may respond differently to certain pain medications, often requiring adjustments in dosages or types of analgesics. Hormonal fluctuations in females can also influence pain perception and treatment efficacy. Additionally, females are more likely to experience anxiety and depression related to chronic pain, affecting their overall pain management [21]. Furthermore, females’ somatosensory homunculus seems to slightly differ from males, and it needs to be fully defined by new studies. Differences in descending pain modulatory systems (with males having a stronger response according to sex and age) and cortex activity (pain unpleasantness related to augmented perigenual anterior cingulate cortex activity in females and decreased ventromedial prefrontal cortex activity in males) have also been described [22,23,24].

Despite the absence of outstanding differences in clinical practice between females and males in response to pain medications, few specific clinical studies have been conducted on sex and gender differences in neuropathic pain. In this real-life clinical study, we investigated the sex and gender-associated clinical differences in efficacy and safety of drugs used to manage subjects affected by neuropathic pain.

## 2. Materials and Methods

### 2.1. Study Design

We carried out a prospective study between 1 February 2021 and 20 April 2024 on subjects with neuropathic pain accessing the Ambulatory of Pain Medicine of “Renato Dulbecco” University Hospital in Catanzaro (Calabria, Italy). The Ethics Committee authorized the study that was conducted in agreement with the Good Clinical Practice guidelines and the Declaration of Helsinki. Our patients signed a written informed consent module before the study started.

### 2.2. Inclusion and Exclusion Criteria

Inclusion criteria: subjects aged over 18 years affected by neuropathic pain and with a ‘Douleur Neuropathique en 4 (DN4) questionnaire’ ≥ 4 were enrolled. Exclusion criteria: clinical signs of nociceptive pain or nociplastic pain; Alzheimer’s disease; or active neoplasm. Moreover, patients with fewer than two clinical accesses to the ambulatory and those who did not sign the informed consent were excluded.

### 2.3. Experimental Protocol

Patients accessing the ambulatory pain medicine for chronic pain were evaluated for neuropathic pain through clinical tests and the DN4 questionnaire. DN4 is a very easy questionnaire consisting of 7 items related to symptoms and 3 related to clinical examination; a total score of 4 out of 10 or more suggests neuropathic pain [25,26].

Patients were enrolled and signed the informed consent. In the context of their clinical access to ambulatory care, comorbidities, demographic data, chronic therapy, drug use and posology, previous ADRs, and pain severity were collected. Pain severity was evaluated through the 11-point numerical rating scale (NRS), where recruited subjects evaluated their pain intensity in a range between 0 and 10 (with 0 representing no pain and 10 an invalidating pain, the highest value on the scale).

Each patient was evaluated during the enrollment (T0) and at the follow-ups at 3 (T1), 6 (T2), and 9 (T3) months. The development of ADRs was evaluated using the Naranjo probability scale, in agreement with our previous studies [27,28,29,30]. Collected data were stored in an Access database with security code protection.

### 2.4. End Points

The primary endpoint was the statistically significant sex and gender-related differences (*p* < 0.05) in change-score (Delta NRS) before and after treatment of neuropathic pain. The secondary endpoint was the statistically significant sex-related differences (*p* < 0.05) in the development of adverse drug reactions, considering comorbidity and polytherapy.

### 2.5. Statistical Analyses

Gaussian continuous variables were described by mean and standard deviation. Median and interquartile range were used in cases of skewness. Counts and percentages were used for categorical variables. The normality distribution of continuous variables was verified by the Shapiro-Wilk test. A T-test was used to compare normally distributed continuous variables between males and females, while the Mann–Whitney test was used in cases of skewness. In cases of low-sized cells (<5), a Chi-squared test or Exact-Fisher test were used. A linear regression model was developed to estimate the influence of gender on the change from baseline in the NRS-score and adjust for all possible confounders. Model building was performed by entering all variables showing a *p*-value lower than 0.250 when analyzed individually and retaining them in the model when the likelihood ratio test for model differences was significant. A second model was developed by propensity score matching with the aim of improving adjustment for all characteristics related to gender. For this purpose, the propensity score was estimated by a binomial logistic model in which sex (male/female) was the independent variable, and the covariate selection was performed as previously described for the ordered logistic model. *p* values < 0.05 were considered statistically significant. Statistical analysis was performed using SPSS 22.0 (International Business Machines Corporation, Armonk, NY, USA) and STATA.16 (www.stata.com) accessed on 21 February 2022 and 13 May 2023.

## 3. Results

### 3.1. Demographic and Clinical Characteristics

**Figure 1:**
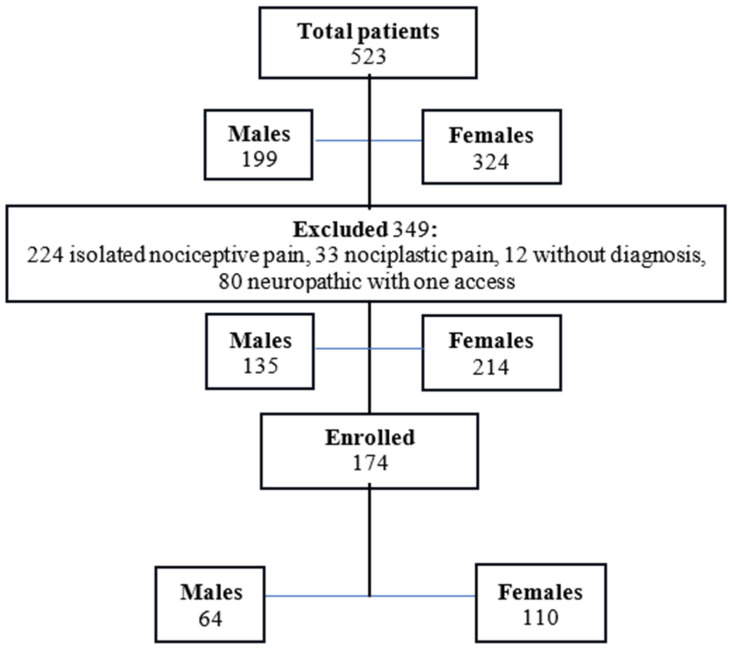
Flow chart showing enrolled patients.

We screened 531 patients (males: 199, age 59.7 ± 11.6; females: 332, mean age 60.3 ± 11.9). In consideration of our recruitment criteria, 174 patients (33.5%, mean age 61.5 ± 13.1; 64 males and 110 females, mean age 60.6 ± 13.4 and 61.96 ± 13.0) affected by neuropathic pain were included (Figure 1 and Table 1).

**Figure 2:**
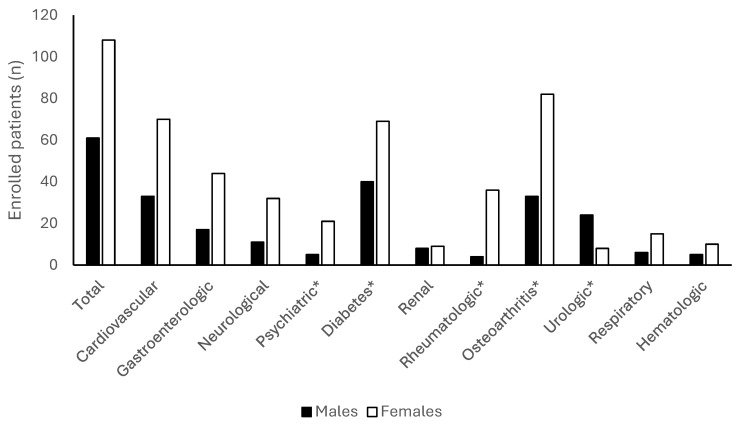
Comorbidity in enrolled patients (n. 174). Data are expressed as absolute values. * *p* < 0.05 between males and females.

Our analysis did not show a significant difference between males and females considering education level, age, and smoking history (*p* > 0.05). Of the 174 recruited subjects, 169 patients (97.1%, mean age 61.8 ± 13.0) had at least one morbidity (males 61, mean age 61.4 ± 13; females 108, mean age 62.1 ± 13.1); osteoarthritis and diabetes were the most frequent (Figure 2). Rheumatologic, psychiatric, and orthopedic diseases were significantly more frequent in females (Figure 2).

The stratification by age showed that 101 patients (38 males and 63 females) were enrolled in the subgroup aged 18–64 years, while 73 (26 males and 47 females) were enrolled in the subgroup aged ≥ 65 years. No significant difference between these groups for BMI, age, DN-4, NRS, smoking, or level of education was observed (Table 2).

Diabetes was the most common comorbidity in the group aged 18–64 years (males 55.3%, females 55.6%, *p* > 0.05) and in elderly males (73.1%), while osteoarthritis and cardiovascular diseases were the most common comorbidities in elderly females (83.0 and 85.1%) (Table 2).

Furthermore, we found a statistically significant variation between males and females for the presence of rheumatological diseases (females’ groups, *p* < 0.01), psychiatric diseases (elderly females group, *p* < 0.01), and urological diseases (elderly males’ group, *p* < 0.01) (Table 2). Each patient used at least one drug for neuropathic pain treatment.

### 3.2. Treatments

#### 3.2.1. Treatments and Sex

In both sexes, the most prescribed drug was pregabalin (46.9% males and 34.5% females) (Table 3). We did not record any significant differences in the prescription of the other drugs (Table 3); nutraceuticals, diamagnetic therapy, and oxygen-ozone therapy were commonly prescribed in both sexes as add-on therapies (Table 3).

Using the Mann–Whitney test, we reported a statistically significant difference (*p* < 0.05) in the dosage of oxycodone in males compared to females (Table 4). The dosages of buprenorphine, fentanyl, tapentadol, duloxetine, and amitriptyline were higher in males than females, without a statistically significant difference (*p* > 0.05).

Concerning the use of acetyl-L-carnitine, 30 patients used a dosage of 500 mg intramuscular, then switched to a 500 mg oral formulation; 5 received the oral formulation, 5 received the intramuscular formulation, and 1 used 1000 mg oral. Sex stratification did not show statistically significant differences between males and females (*p* > 0.05).

#### 3.2.2. Drug Treatment and BMI

The relationship between treatments and BMI stratified by sex did not highlight significant differences (Table 5). Duloxetine, which, in females, was used more frequently in obese patients (BMI > 30; Table 5), did not reach statistical significance (*p* = 0.056).

#### 3.2.3. Treatments and Age

Among males aged under 65 years, the use of opioids and diamagnetic therapy was prevalent, with no discernible difference when compared to females (Table 6). Only young patients of both sexes received fentanyl. The rates of use of amitriptyline and duloxetine were greater in females, but they did not achieve statistical significance in older females’ cases.

#### 3.2.4. Treatments and Safety

During the study, 32 patients (18.4%), 19 females (17.3%), and 13 males (20.3%) developed ADRs (Table 7).

### 3.3. Pain Evaluation

Data recorded during admission (T0) and at the end of the study (T3) documented a significant improvement in pain (*p* < 0.01) without differences between males and females (Table 8).

The linear regression model highlights how in women, the delta NRS is higher than men by 0.37 (95% CI: −0.34, 1.09), thus indicating greater treatment efficacy without reaching statistical significance. The model suggests potential positive predictors such as the DN4 value, with a statistically significant efficacy increase of 0.28 points (95% CI: 0.04, 0.52) per one-point increase in DN4, treatment with buprenorphine (coeff: 1.53; 95% CI: 0.32, 2.75), and tramadol (coeff: 0.94; 95% CI: 0.10, 1.79). In contrast, ozone treatment emerges as a negative predictor of delta NRS (coeff: −1.14; 95% CI: −1.88, −0.40). Treatment with codeine (coeff: 0.69; 95% CI: −0.07, 1.45), presence of cardiovascular (coeff: 0.50; 95% CI: −0.20, 1.20), and psychiatric (coeff: 0.82; 95% CI: −0.10, 1.73) pathologies do not significantly correlate with delta NRS. The difference in treatment efficacy between men and women was confirmed by propensity-score matching analysis with a coefficient of 0.41 (95% CI: −0.73, 1.12). No significant influence of gender-related factors such as smoking and educational level was found (Table 9).

## 4. Discussion

In this clinical study performed in a real-life setting, we evaluated the role of sex in the management of neuropathic pain and documented that females are commonly affected by neuropathic pain without any correlation with economic status or education level. A previous study in diabetic patients with chronic pain documented that females have lower levels of education compared to males of the same age and same health status, supporting a lower quality of life than males [31]. In contrast, in our study we did not record any sex-related variation in economic status and quality of life.

In our study, we documented that, among comorbidities, diabetes and urological diseases were more common in elderly males with chronic neuropathic pain, while cardiovascular, rheumatological diseases, osteoarthritis, and psychiatric diseases were more common in elderly females. Previous data showed that females have a higher incidence of autoimmune susceptibility, neurodegenerative disease, back pain, and migraine in clinical settings [32,33].

Recent studies [34] documented that neuropathic pain is associated with psychiatric comorbidities affecting quality of life. Our patients with psychiatric comorbidities were mainly affected by an anxious-depressive disorder. The interplay between anxiety, depression, and pain is complex, since pain may determine the onset of symptoms related to these psychiatric conditions. Conversely, anxiety and depression may determine pain symptoms or worsen an existing clinical condition. Several correlations have been observed, including the involvement of similar brain areas (e.g., insular cortex, thalamus, amygdala), the presence of neuroinflammation (with studies highlighting the role of cytokines in the comorbidity between pain and mood disorders), the presence of similar chronification patterns at imaging and of common clinical patterns (sleep disturbance, the impossibility of an objective measurement of symptoms), and lastly, the possible experience of social exclusion, stigma, and invalidating interactions. Furthermore, the management of the two conditions is generally more difficult in cases of co-occurrence [35,36]. In this paper, we observe psychiatric conditions as one of the most frequent comorbidities. Curiously, in our multivariate linear model, we observed a positive correlation between psychiatric diagnosis and the increase of delta NRS (associated with a better clinical outcome), although without reaching statistical significance. This result may be justified by the need of the feeling by these subjects of a complete take of charge by the physicians. Nevertheless, the relapse rate is generally very high.

Finally, in agreement with a recent clinical study [37], we did not report any association between kidney diseases and neuropathic pain.

The prescription/deprescribing of drugs for pain management in these patients requires a deep knowledge of each formulation’s characteristics (e.g., accumulation, metabolism, possibility of dialysis, and drug-drug and drug-disease interactions). Considering the reduced number of pharmacokinetic studies in subjects with ESRD, the therapeutic range and the risk of starting/continuing a pharmacologic treatment should be evaluated scrupulously in the therapeutic algorithm, associating patients’ follow-up and therapeutic drug monitoring (TDM), if necessary [38].

Boorman and Keay [39], in an experimental model, showed that the morphine response was greater in males than females and that males develop tolerance sooner. These findings reflect the results of some human clinical studies in which females required higher doses [40] and males de-escalate faster than females [41].

However, these data are contrary to our results, considering that males had significantly higher medium doses of oxycodone, and, despite other opioids not reaching statistical significance, dosages between males and females were similar or higher for males (except tramadol, which was higher in females).

In agreement with previous studies [4,14,42], our results show that a multi-modal treatment is an effective strategy in reducing pain in males and females.

Despite not being properly neuropathic pain medications, muscle relaxants are very useful in improving pain perception in patients with neuropathic cervicobrachial or low back pain [43,44].

In the group of other therapies, we recorded a higher rate of treatment with diamagnetic therapy, oxygen-ozone therapy, and acetyl-L-carnitine in males. In contrast, for females, nutraceuticals were commonly prescribed. None of these results were statistically significant. Considering the higher rate of comorbidity in females, we would expect a more frequent use of oxygen-ozone, and diamagnetic therapy, considering their safer safety profile in comparison to drugs. In fact, these treatments, added to the common drugs, resulted in a decrease in dosage consumption [4,14,18,45]. In our study, multimodal therapy was commonly used in males compared to females. Nevertheless, the higher percentage of nutraceuticals used in females may indicate the necessity of using natural products with fewer side effects [46] to synergistically empower the effect of drugs or to reduce their usage.

### 4.1. Drug Prescription

Concerning drug prescription according to BMI, only duloxetine was consumed more frequently in females with obesity. The correlation between depression and obesity is commonly reported [47,48]; this could also be related to the chronic use of SSRI [49], even if in our study we did not record this use. Pregabalin was prescribed in patients with increased BMI in both sexes because, as described, obesity is related to increased levels of pain due to mechanical and cytokine mechanisms [50,51].

### 4.2. Age

Age sub-analysis did not highlight any statistically significant difference. However, males received opioids before 65 years, whereas females received them in advanced age. Weak opioids (generally used for brief time intervals) were prescribed above all in younger patients in both sexes (except for codeine in females, which was nearly balanced), as a chronic treatment would be more difficult to tolerate due to impairment in their everyday life [52].

Amitriptyline was used more in advanced age for males and young age for females, whereas acetyl-L-carnitine showed an opposite trend.

Interestingly, nutraceuticals were preferred in advanced age, probably for their safety and reduced number of interactions [46]. Conversely, diamagnetic therapy was prescribed in the younger group, maybe holding in account the difficulty of bringing advanced age patients into the hospital to receive the treatment. The retrospective study by Freburger and Holmes [53], on 38,312 people ≥65 years, evidenced an inverse correlation between physical therapy and age. Other factors such as income and living in a metropolitan area were positively associated with the rate of physical therapy. Furthermore, the most important contraindications of diamagnetic therapy (e.g., severe cardiopathies, cancer, pacemaker, or the presence of metallic parts) [54] are more likely in advanced age.

Drug dosage was generally similar between sexes or higher in males, except for oxycodone, which was higher in males. In general, females were estimated to necessitate a higher opioid dose than males [40], even though they have been reported to need a lower dose for postoperative pain [55]. We found no data that makes a comparison in this sense concerning duloxetine, amitriptyline, and pregabalin.

In our study we did not record the development of severe adverse drug reactions, except for 2 cases of hypertensive peak (buprenorphine in the first case; tramadol plus PEA, alpha-lipoic acid, and acetyl-L-carnitine nutraceutical in the second case) and a case of hypersensitivity (cyclobenzaprine). The adverse events were described in drug labels, showing mild CNS effects such as drowsiness, confusion, and headache (especially for pregabalin, which was also the most administered drug) [56,57,58]. Acetyl-L-carnitine and diamagnetic therapy showed no significant ADRs, with optimal safety, in agreement with our experience and available evidence [14,59]. Tapentadol had no side effects, probably due to the low number of treated patients, but also considering its optimal safety profile on gastrointestinal effects in comparison to oxycodone [43]. No significant difference related to sex was observed. It is interesting to note that although females are generally expected to experience side effects [60], in our group they had fewer side effects compared to males (17.3% vs. 20.3%).

Previously, Khan et al. [61] revealed that smoking impaired clinical symptoms in patients with neuropathic pain. In our study, we did not record any association between smoking and neuropathic pain. This effect is probably related to the low prevalence of smoking in the enrolled patients.

## 5. Conclusions

No significant differences in NRS change score were found according to sex and other gender factors such as instruction level and smoking, despite a slightly greater clinical benefit being found in females with neuropathic pain. No significant differences in safety outcomes were found, despite females exhibiting a minor number of adverse events in comparison to males. Little differences concerning drug prescription and drug dosage were found.

## 6. Limitations

There were several limitations in this study. Firstly, our court is relatively small to obtain definitive conclusions, and the total of females is high if compared to males. However, the real-life setting shows a higher number of females accessing our unit. Data were collected in a clinical room of pain medicine where a clinical pharmacologist was the main responsible for the diagnostic and therapeutic processes. In consideration of this fact, the evaluation by a specialist probably decreased the development of side effects and DDI, holding also in account polytherapy, comorbidity, smoke, and educational level.

In conclusion, our prospective study in adult patients diagnosed with neuropathic pain showed that sex and gender factors do not play a role in the effect of the treatment in patients with neuropathic pain.

## Figures and Tables

**Table 1:** Demographic characteristics at enrollment (n,%) (males: 64; females: 110).

	Males n: 64	%	Females n: 110	%
**Age**				
18–64	38	59.4	63	57.3
≥65	26	40.6	47	42.7
**Degree**				
Yes	12	18.8	24	21.8
No	52	81.2	86	78.2
**Body mass index**				
<25	16	25.0	34	30.9
25–30	33	51.6	37	33.6
≥30	15	23.4	39	35.5
**Smokers**				
Yes (or former smokers)	37	57.8	36	32.7
No	27	42.2	74	67.3
**Diagnosis**				
Lumbar radicular pain	25	39.1	52	47.3
Lumbar radicular pain + cervical radiculopathy	26	40.6	50	45.5
Cervical radiculopathy	6	9.4	6	5.5
Facial pain	3	4.7	0	0
Phantom limb pain	2	3.1	0	0
Back pain	1	1.6	0	0
Lumbar radicular pain + diabetic neuropathy	0	0	1	1.9
Diabetic neuropathy	1	1.6	0	0
Lumbar radicular pain + cervical radiculopathy + facial pain	0	0	1	0.9

**Table 2:** Characteristics of enrolled patients (males 64, females 110) stratified for age. BMI: Body mass index; DN4: Douleur Neuropathique en 4 Questions. NRS: numerical rating scale. ** *p* < 0.05.

	Overall *p* (Differences between Age Classes)	18–64		≥65		Within Sex *p*	18–64		≥65		Within Sex *p*
		Males					Females				
		Number	Percentage	Number	Percentage		Number	Percentage	Number	Percentage	
Enrolled		38	59.4	26	40.6		63	57.3	47	42.7	
Age	0.000 **	52.1 ± 9.8		73.0 ± 6.0		0.000 **	53.4 ± 9.4		73.4 ± 6.9		0.000 **
BMI	0.478	27.4 ± 3.9		26.9 ± 4.7		0.614	27.6 ± 5.5		28.8 ± 5.0		0.267
Degree	0.734	6	15.8	6	23.1	0.463	14	22.2	10	21.3	0.905
Smokers	0.845	20	52.6	17	65.4	0.310	23	36.5	13	27.7	0.328
		Median	IQR	Median	IQR		Median	IQR	Median	IQR	
NRS	0.436	8	3	8	2	0.303	8	1	9	2	0.651
DN4	0.503	5	1	5	1	0.628	5	2	5.5	1	0.356
Comorbidities											
		Number	Percentage	Number	percentage		Number	Percentage	Number	percentage	
Comorbidities (overall)	0.054	35	92.1	26	100.0	0.142	61	96.8	47	100.00	0.218
Cardiovascular diseases	0.000 **	16	42.1	17	65.4	0.067	31	49.2	39	83.0	0.000 **
Diabetes	0.021 **	21	55.3	19	73.1	0.148	35	55.6	34	72.3	0.072
Osteoarthritis	0.005 **	16	42.1	17	65.4	0.067	42	66.7	40	85.1	0.028 **
Urologic diseases	0.003 **	8	21.1	16	61.5	0.001 **	3	4.8	5	10.6	0.283
Gastrointestinal diseases	0.438	8	21.1	9	34.6	0.228	25	39.7	19	40.4	0.937
Neurological diseases	0.989	5	13.2	6	23.1	0.302	20	31.7	12	25.5	0.478
Rheumatological diseases	0.035 **	3	7.9	1	3.8	0.640	26	41.3	10	21.3	0.027 **
Psychiatric diseases	0.210	5	13.2	0	0.0	0.074	13	20.6	8	17.0	0.633
Renal diseases	0.045 **	4	10.5	4	15.4	0.705	2	3.2	7	14.9	0.036 **
Respiratory diseases	0.133	2	5.3	4	15.4	0.213	7	11.1	8	17.0	0.372
Hematological diseases	0.350	1	2.6	4	15.4	0.149	6	9.5	4	8.5	1.000

**Table 3:** Drug used in enrolled patients (n = 174) stratified for sex (number of patients and percentage) ** *p* < 0.05.

Drugs	Males n: 64	%	Females n: 110	%	*p*
**Opioids**					
Oxycodone/naloxone	8	12.5	8	7.3	0.250
Oxycodone	5	7.8	4	3.6	0.292
Buprenorphine	3	4.7	10	9.1	0.378
Codeine	10	15.6	24	21.8	0.320
Tramadol	13	20.3	15	13.6	0.248
Fentanyl	2	3.1	2	1.8	0.626
Tapentadol	2	3.1	3	2.7	1.000
**Antidepressants**					
Amitriptyline	5	7.8	9	8.2	0.931
Duloxetine	10	15.6	18	16.4	0.898
**Antiepileptics**					
Pregabalin	30	46.9	38	34.5	0.108
**Myorelaxants**					
Eperisone	12	18.8	19	17.3	0.806
Cannabidiol and β-caryophyllene	8	12.5	9	8.2	0.355
Cyclobenzaprine	5	7.8	9	8.2	0.931
Tizanidine	2	3.1	4	3.6	1.000
**Other treatments**					
L-acetyl-carnitine	18	28.1	23	20.9	0.279
Nutraceuticals	35	54.7	64	58.2	0.654
Diamagnetic therapy	28	43.8	42	38.2	0.470
Oxygen-ozone therapy	46	71.9	73	66.4	0.451
Capsaicin cream	3 **	4.7	0	0	0.048
Lidocaine	1	1.6	1	0.9	1.000
Gabapentin	1	1.6	0	0	0.368
Antipsychotics	1	1.6	2	1.8	1.000
Facet joint injections	1	0.9	1	1.6	1.000

The bold is useful to highlight each drug class.

**Table 4:** Drug treatment and dosage used in enrolled patients (males: 64, females: 110). Gaussian continuous variables were described by the mean and the standard deviation. Median and interquartile range were used in case of skewness. * *p* < 0.05.

Drug Treatment	Dosage in Males	Dosage in Females	*p*
Oxycodone/naloxone (mg)	17.50 (23.8)	13.75 (32.5)	1.000
Buprenorphine (µg/h)	35.0 (-)	5.0 (15.0)	0.750
Codeine (mg)	30.0 (30.0)	30.0 (22.5)	0.446
Fentanyl (µg/h)	62.50 ± 17.67	37.50 ± 17.68	0.293
Tramadol (mg)	75.0 (43.8)	75.0 (25.0)	0.711
Oxycodone (mg)	30.0 (70.0) *	5.0 (11.3)	0.033
Tapentadol (mg)	225.00 ± 106.06	108.33 ± 80.36	0.250
Duloxetine (mg)	30.0 (30.0)	30.0 (7.5)	0.327
Amitriptyline (mg)	12.0 (27.0)	10.0 (5.0)	0.176
Pregabalin (mg)	126.66 ± 94.89	121.71 ± 66.06	0.801
Tizanidine (mg)	4.0 (−)	2.0 (1.5)	0.114

**Table 5:** Drug used in males and females, considering the body mass index (BMI).

Treatment	Sex	BMI							
		<25		25–30		≥30			
		Number	%	Number	%	Number	%	Within Sex *p*	Overall *p*
**Opioids**									
Oxycodone/naloxone	Males	1	6.3	6	18.2	1	6.7	0.412	0.269
	Females	1	2.9	3	8.1	4	10.3	0.532	
Oxycodone	Males	0	0.0	5	15.2	0	0.0	0.067	0.569
	Females	1	2.9	0	0.0	3	7.7	0.265	
Buprenorphine	Males	1	6.3	2	6.1	0	0.0	1.000	0.446
	Females	3	8.8	5	13.5	2	5.1	0.454	
Codeine	Males	2	12.5	4	12.1	4	26.7	0.397	0.348
	Females	10	29.4	6	16.2	8	20.5	0.393	
Tramadol	Males	3	18.8	5	15.2	5	33.3	0.391	0.381
	Females	2	5.9	8	21.6	5	12.8	0.180	
Fentanyl	Males	0	0.0	2	6.1	0	0.0	1.000	0.562
	Females	0	0.0	0	0.0	2	5.1	0.328	
Tapentadol	Males	0	0.0	2	6.1	0	0.0	1.000	0.227
	Females	0	0.0	2	5.4	1	2.6	0.646	
**Antidepressants**									
Amitriptyline	Males	1	6.3	4	12.1	0	0.0	0.587	0.687
	Females	3	8.8	3	8.1	3	7.7	1.000	
Duloxetine	Males	2	12.5	5	15.2	3	20.0	0.816	0.060
	Females	4	11.8	3	8.1	11	28.2	0.056	
**Antiepileptics**									
Pregabalin	Males	5	31.3	17	51.5	8	53.3	0.349	0.076
	Females	8	23.5	15	40.5	15	38.5	0.262	
**Myorelaxants**									
Eperisone	Males	3	18.8	6	18.2	3	20.0	1.000	0.924
	Females	5	14.7	7	18.9	7	17.9	0.887	
CBD and β-caryophyllene	Males	2	12.5	3	9.1	3	20.0	0.547	0.589
	Females	2	5.9	6	16.2	1	2.6	0.103	
Cyclobenzaprine	Males	1	6.3	3	9.1	1	6.7	1.000	0.384
	Females	1	2.9	5	13.5	3	7.7	0.289	
Tizanidine	Males	0	0.0	2	6.1	0	0.0	1.000	0.285
	Females	0	0.0	2	5.4	2	5.1	0.545	
**Other treatments**									
L-acetyl-carnitine	Males	4	25.0	10	30.3	4	26.7	1.000	0.782
	Females	8	23.5	8	21.6	7	17.9	0.836	
Nutraceuticals	Males	11	68.8	17	51.5	7	46.7	0.407	0.251
	Females	18	52.9	18	48.6	28	71.8	0.094	
Diamagnetic therapy	Males	7	43.8	15	45.5	6	40.0	0.940	0.137
	Females	17	50.0	15	40.5	10	25.6	0.095	
Oxygen-ozone therapy	Males	11	68.8	25	75.8	10	66.7	0.769	0.109
	Females	19	55.9	29	78.4	25	64.1	0.125	

The bold is useful to highlight each drug class.

**Table 6:** Drug used in enrolled patients (males = 64, females = 110) stratified by age. Subgroup 18–64 (m = 38, f = 63); subgroup ≥ 65 years (m = 26, f = 47).

	Sex	Age					
		18–64		≥65			
		Number	Percentage	Number	Percentage	Within Sex *p*	Overall *p*
**Opioids**							
Oxycodone/naloxone	Males	6	15.8	2	7.7	0.456	0.879
	Females	3	4.8	5	10.6	0.283	
Oxycodone	Males	2	5.3	3	11.5	0.389	0.494
	Females	2	3.2	2	4.3	1.000	
Buprenorphine	Males	3	7.9	0	0.0	0.265	0.561
	Females	6	9.5	4	8.5	1.000	
Codeine	Males	7	18.4	3	11.5	0.510	0.918
	Females	13	20.6	11	23.4	0.728	
Tramadol	Males	9	23.7	4	15.4	0.534	0.251
	Females	10	15.9	5	10.6	0.429	
Fentanyl	Males	2	5.3	0	0.0	0.510	0.140
	Females	2	3.2	0	0.0	0.506	
Tapentadol	Males	1	2.6	1	3.8	1.000	0.651
	Females	1	1.6	2	4.3	0.575	
**Antidepressants**							
Amitriptyline	Males	2	5.3	3	11.5	0.389	0.943
	Females	6	9.5	3	6.4	0.730	
Duloxetine	Males	6	15.8	4	15.4	1.000	0.755
	Females	11	17.5	7	14.9	0.719	
**Antiepileptics**							
Pregabalin	Males	19	50.0	11	42.3	0.545	0.882
	Females	20	31.7	18	38.3	0.475	
**Muscle relaxants**							
Eperisone	Males	7	18.4	5	19.2	0.935	0.690
	Females	10	15.9	9	19.1	0.653	
Cannabidiol and β-caryophyllene	Males	5	13.2	3	11.5	1.000	0.653
	Females	4	6.3	5	10.6	0.493	
Cyclobenzaprine	Males	3	7.9	2	7.7	1.000	0.400
	Females	7	11.1	2	4.3	0.296	
Tizanidine	Males	2	5.3	0	0.0	0.510	0.403
	Females	3	4.8	1	2.1	0.634	
**Other treatments**							
L-acetyl-carnitine	Males	12	31.6	6	23.1	0.457	0.772
	Females	11	17.5	12	25.5	0.303	
Nutraceuticals	Males	20	52.6	15	57.7	0.690	0.444
	Females	35	55.6	29	61.7	0.518	
Diamagnetic therapy	Males	20	52.6	8	30.8	0.083	0.093
	Females	26	41.3	16	34.0	0.440	
Oxygen-ozone therapy	Males	27	71.1	19	73.1	0.860	0.760
	Females	43	68.3	30	63.8	0.627	

Bold is useful to highlight each drug class.

**Table 7:** Types of adverse drug reactions (ADRs) recorded in treated patients (males 13, females 19) for the management of neuropathic pain. * Same patient with more ADRs during polytherapy. Females’ groups *a, *b, *c, *d, *e, *f, represent six patients (a–f) that developed more than one ADR.

	Males (n: 13)			Females (n: 19)			*p*
	Number	%	Type	Number	%	Type	
Oxycodone	1	7.7	Stypsis (1)	1	5.3	Drowsiness (1) *a	1.000
oxycodone/naloxone	2	15.4	stypsis (1) *; confusion (1)	1	5.3	stypsis (1) *f	1.000
buprenorphine	1	7.7	blood hypertension (1)	2	10.5	stypsis (1); skin rash (1) *a	1.000
Codeine	1	7.7	Stypsis (1)	1	5.3	stypsis (1) *b	1.000
Tramadol	0	0.0		2	10.5	blood hypertension (1), (1) *c	0.535
Tapentadol	0	0.0		0	0.0		N.C.
Fentanyl	1	7.7	Stypsis (1)	0	0.0		0.364
amitriptyline	2	15.4	confusion (1); drowsiness (1)	1	5.3	Confusion	0.299
Duloxetine	1	7.7	Confusion (1)	3	15.8	confusion (1); drowsiness (2)	1.000
Pregabalin	2	15.4	confusion (1) *; drowsiness (1)	6	31.6	Drowsiness (3), (1) *d, (1) *e, (1) *f	0.712
cyclobenzaprine	4	30.8	drowsiness (3), (1) *	4	21.1	Drowsiness (1), (1) *b; (1) *d; skin rash (1) *e	1.000
Nutrients	0	0.0		3	15.8	blood hypertension (1) *c; bowel dysfunction (1), (1) *f	0.555
oxygen-ozone therapy	0	0.0		2	10.5	pain in the site of administration (2)	0.535

N.C.: It’s “not calculable” since no side effects were observed with tapentadol.

**Table 8:** Pain evaluation in males and females. NRS: numerical rating scale.

	Admission	End of the Study	*p*
NRS			
Males	8.0 (2.8)	5.0 (4.0)	<0.01
Females	8.0 (2.0)	5.0 (4.0)	<0.01
	*p* > 0.05	*p* > 0.05	

**Table 9:** Linear modeling of NRS-change score. Estimated by the multiple linear regression model. R-squared: 0.17. Linearity, homoscedasticity, and normality of residuals were verified.

Delta NRS	Coefficient	[95% Conf. Interval]		*p*
Sex	0.37	−0.34	1.09	0.301
DN4	0.28	0.04	0.52	0.023
Cardiovascular comorbidities	0.50	−0.20	1.20	0.163
Psychiatric comorbidities	0.82	−0.10	1.73	0.081
Buprenorphine	1.53	0.32	2.75	0.014
Codeine	0.69	−0.07	1.45	0.075
Tramadol	0.94	0.10	1.79	0.029
Oxygen-ozone therapy	−1.14	−1.88	−0.40	0.003
NRS first access	−0.37	−0.62	−0.12	0.004

## Data Availability

The data presented in this study are available on request from the corresponding author due to privacy and ethical restrictions.

## References

[1] Cohen S.P., Vase L., Hooten W.M. (2021). Series Chronic Pain 1 Chronic pain: An update on burden, best practices, and new advances. Lancet.

[2] Baron R., Binder A., Wasner G. (2010). Neuropathic pain: Diagnosis, pathophysiological mechanisms, and treatment. Lancet Neurol..

[3] IASP International Association for the Study of Pain-Terminology. https://www.iasp-pain.org/resources/terminology/.

[4] Marcianò G., Vocca C., Evangelista M., Palleria C., Muraca L., Galati C., Monea F., Sportiello L., De Sarro G., Capuano A. (2023). The Pharmacological Treatment of Chronic Pain: From Guidelines to Daily Clinical Practice. Pharmaceutics.

[5] Attal N., Bouhassira D. (2019). Translational neuropathic pain research. Pain.

[6] Cavalli E., Mammana S., Nicoletti F., Bramanti P., Mazzon E. (2019). The neuropathic pain: An overview of the current treatment and future therapeutic approaches. Int. J. Immunopathol. Pharmacol..

[7] Clayton A., Kornstein S., Prakash A., Mallinckrodt C., Wohlreich M. (2007). Changes in sexual functioning associated with duloxetine, escitalopram, and placebo in the treatment of patients with major depressive disorder. J. Sex. Med..

[8] Bozkurt M., Gocmez C., Soylemez H., Daggulli M., Em S., Yildiz M., Atar M., Bozkurt Y., Ozbey I. (2014). Association between neuropathic pain, pregabalin treatment, and erectile dysfunction. J. Sex. Med..

[9] Kamper D. (2022). Palmitoylethanolamide (PEA) in the treatment of neuropathic pain: A case study. Nutr. Health.

[10] Viana M.D.M., Lauria P.S.S., de Lima A.A., Opretzka L.C.F., Marcelino H.R., Villarreal C.F. (2022). Alpha-Lipoic Acid as an Antioxidant Strategy for Managing Neuropathic Pain. Antioxidants.

[11] Sarzi-Puttini P., Giorgi V., Di Lascio S., Fornasari D. (2021). Acetyl-L-carnitine in chronic pain: A narrative review. Pharmacol. Res..

[12] Chirchiglia D., Paventi S., Seminara P., Cione E., Gallelli L. (2018). N-Palmitoyl Ethanol Amide Pharmacological Treatment in Patients With Nonsurgical Lumbar Radiculopathy. J. Clin. Pharmacol..

[13] Chirchiglia D., Chirchiglia P., Marotta R., Gallelli L. (2019). Add-on administration of ultramicronized palmitoylethanolamide in the treatment of new-onset burning mouth syndrome. Int. Med. Case Rep. J..

[14] Pullano S.A., Marcianò G., Bianco M.G., Oliva G., Rania V., Vocca C., Cione E., De Sarro G., Gallelli L., Romeo P. (2022). FT-IR Analysis of Structural Changes in Ketoprofen Lysine Salt and KiOil Caused by a Pulsed Magnetic Field. Bioengineering.

[15] Premi E., Benussi A., La Gatta A., Visconti S., Costa A., Gilberti N., Cantoni V., Padovani A., Borroni B., Magoni M. (2018). Modulation of long-term potentiation-like cortical plasticity in the healthy brain with low frequency-pulsed electromagnetic fields. BMC Neurosci..

[16] Ahmed Z., Wieraszko A. (2015). Pulsed magnetic stimulation modifies amplitude of action potentials in vitro via ionic channels-dependent mechanism. Bioelectromagnetics.

[17] Roberti R., Marcianò G., Casarella A., Rania V., Palleria C., Muraca L., Citraro R., De Sarro G., Serra R., Romeo P. (2022). High-Intensity, Low-Frequency Pulsed Electromagnetic Field as an Odd Treatment in a Patient with Mixed Foot Ulcer: A Case Report. Reports.

[18] Roberti R., Marcianò G., Casarella A., Rania V., Palleria C., Vocca C., Catarisano L., Muraca L., Citraro R., Romeo P. (2022). Diamagnetic Therapy in a Patient with Complex Regional Pain Syndrome Type I and Multiple Drug Intolerance: A Case Report. Reports.

[19] WHO World Health Organization Gender. https://www.who.int/health-topics/gender#tab=tab_1.

[20] Abraham A., Barnett C., Katzberg H.D., Lovblom L.E., Perkins B.A., Bril V. (2018). Sex differences in neuropathic pain intensity in diabetes. J. Neurol. Sci..

[21] Zhang L., Losin E.A.R., Ashar Y.K., Koban L., Wager T.D. (2021). Gender Biases in Estimation of Others’ Pain. J. Pain.

[22] Di Noto P.M., Newman L., Wall S., Einstein G. (2013). The hermunculus: What is known about the representation of the female body in the brain? Cereb. Cortex.

[23] Girard-Tremblay L., Auclair V., Daigle K., Léonard G., Whittingstall K., Goffaux P. (2014). Sex differences in the neural representation of pain unpleasantness. J. Pain.

[24] Failla M.D., Beach P.A., Atalla S., Dietrich M.S., Bruehl S., Cowan R.L., Monroe T.B. (2024). Gender Differences in Pain Threshold, Unpleasantness, and Descending Pain Modulatory Activation Across the Adult Life Span: A Cross Sectional Study. J. Pain.

[25] Bouhassira D., Attal N., Alchaar H., Boureau F., Brochet B., Bruxelle J., Cunin G., Fermanian J., Ginies P., Grun-Overdyking A. (2005). Comparison of pain syndromes associated with nervous or somatic lesions and development of a new neuropathic pain diagnostic questionnaire (DN4). Pain.

[26] Truini A., Galosi E., Zanette G., Di Stefano G., Raffaeli W., Magrinelli F. (2017). The Italian version of the DN4 questionnaire for differential diagnosis of neuropathic pain. Pain Pract..

[27] Caroleo B., Migliore A., Cione E., Zampogna S., Perticone F., De Sarro G., Gallelli L. (2019). Double Infection in a Patient with Psoriatic Arthritis Under TNF-alpha Blockers Therapy: A Case Report. Curr. Drug Saf..

[28] Gallelli L., Ferreri G., Colosimo M., Pirritano D., Guadagnino L., Pelaia G., Maselli R., De Sarro G.B. (2002). Adverse drug reactions to antibiotics observed in two pulmonology divisions of Catanzaro, Italy: A six-year retrospective study. Pharmacol. Res..

[29] Gallelli L., Colosimo M., Pirritano D., Ferraro M., De Fazio S., Marigliano N.M., De Sarro G. (2007). Retrospective evaluation of adverse drug reactions induced by nonsteroidal anti-inflammatory drugs. Clin. Drug Investig..

[30] Gallelli L., Nardi M., Prantera T., Barbera S., Raffaele M., Arminio D., Pirritano D., Colosimo M., Maselli R., Pelaia G. (2004). Retrospective analysis of adverse drug reactions induced by gemcitabine treatment in patients with non-small cell lung cancer. Pharmacol. Res..

[31] Dermanovic Dobrota V., Hrabac P., Skegro D., Smiljanic R., Dobrota S., Prkacin I., Brkljacic N., Peros K., Tomic M., Lukinovic-Skudar V. (2014). The impact of neuropathic pain and other comorbidities on the quality of life in patients with diabetes. Health Qual. Life Outcomes.

[32] Coraggio V., Guida F., Boccella S., Scafuro M., Paino S., Romano D., Maione S., Luongo L. (2018). Neuroimmune-driven neuropathic pain establishment: A focus on gender differences. Int. J. Mol. Sci..

[33] Ghazisaeidi S., Muley M.M., Salter M.W. (2023). Neuropathic Pain: Mechanisms, Sex Differences, and Potential Therapies for a Global Problem. Annu. Rev. Pharmacol. Toxicol..

[34] Nicholson B., Verma S. (2004). Comorbidities in Chronic Neuropathic Pain. Pain Med..

[35] De La Rosa J.S., Brady B.R., Ibrahim M.M., Herder K.E., Wallace J.S., Padilla A.R., Vanderah T.W. (2024). Co-occurrence of chronic pain and anxiety/depression symptoms in U.S. adults: Prevalence, functional impacts, and opportunities. Pain.

[36] Vieira W.F., Coelho D.R.A., Litwiler S.T., McEachern K.M., Clancy J.A., Morales-Quezada L., Cassano P. (2024). Neuropathic pain, mood, and stress-related disorders: A literature review of comorbidity and co-pathogenesis. Neurosci. Biobehav. Rev..

[37] Kitala-Tańska K., Kania-Zimnicka E., Tański D., Kwella N., Stompór T., Stompór M. (2024). Prevalence and Management of Chronic Pain, Including Neuropathic Pain, in Dialysis Patients with End-Stage Renal Disease. Med. Sci. Monit..

[38] Raouf M., Bettinger J., Wegrzyn E.W., Mathew R.O., Fudin J.J. (2020). Pharmacotherapeutic Management of Neuropathic Pain in End-Stage Renal Disease. Kidney Dis..

[39] Boorman D.C., Keay K.A. (2022). Sex differences in morphine sensitivity are associated with differential glial expression in the brainstem of rats with neuropathic pain. J. Neurosci. Res..

[40] Dahan A., Kest B., Waxman A.R., Sarton E. (2008). Sex-specific responses to opiates: Animal and human studies. Anesth. Analg..

[41] Kaplovitch E., Gomes T., Camacho X., Dhalla I.A., Mamdani M.M., Juurlink D.N. (2015). Sex differences in dose escalation and overdose death during chronic opioid therapy: A population-based cohort study. PLoS ONE.

[42] Rania V., Marcianò G., Casarella A., Vocca C., Palleria C., Calabria E., Spaziano G., Citraro R., De Sarro G., Monea F. (2023). Oxygen–Ozone Therapy in Cervicobrachial Pain: A Real-Life Experience. J. Clin. Med..

[43] Colorado Division of Workers’ Compensation (2017). Chronic Pain Disorder Medical Treatment Guideline.

[44] See S., Ginzburg R. (2008). Choosing a skeletal muscle relaxant. Am. Fam. Physician.

[45] Bocci V. (2011). Ozone: A New Medical Drug.

[46] Ilari S., Proietti S., Russo P., Malafoglia V., Gliozzi M., Maiuolo J., Oppedisano F., Palma E., Tomino C., Fini M. (2022). A Systematic Review and Meta-Analysis on the Role of Nutraceuticals in the Management of Neuropathic Pain in In Vivo Studies. Antioxidants.

[47] Milaneschi Y., Simmons W.K., van Rossum E.F.C., Penninx B.W. (2019). Depression and obesity: Evidence of shared biological mechanisms. Mol. Psychiatry.

[48] Jantaratnotai N., Mosikanon K., Lee Y., McIntyre R.S. (2017). The interface of depression and obesity. Obes. Res. Clin. Pract..

[49] Narouze S., Souzdalnitski D. (2015). Obesity and chronic pain: Systematic review of prevalence and implications for pain practice. Reg. Anesth. Pain Med..

[50] Zhou J., Mi J., Peng Y., Han H., Liu Z. (2021). Causal Associations of Obesity With the Intervertebral Degeneration, Low Back Pain, and Sciatica: A Two-Sample Mendelian Randomization Study. Front. Endocrinol..

[51] Zhang D.-H., Fan Y.-H., Zhang Y.-Q., Cao H. (2023). Neuroendocrine and neuroimmune mechanisms underlying comorbidity of pain and obesity. Life Sci..

[52] Benyamin R., Trescot A.M., Datta S., Buenaventura R., Adlaka R., Sehgal N., Glaser S.E., Vallejo R. (2008). Opioid complications and side effects. Pain Physician.

[53] Freburger J.K., Holmes G.M. (2005). Physical therapy use by community-based older people. Phys. Ther..

[54] PERISO CTU MEGA 20. https://periso.ch/wp-content/uploads/2019/11/MOD07-2-5-CTU-Mega-20-IFU-ENG-web.pdf.pdf.

[55] Pieretti S., Di Giannuario A., Di Giovannandrea R., Marzoli F., Piccaro G., Minosi P., Aloisi A.M. (2016). Gender differences in pain and its relief. Ann. Ist. Super Sanità.

[56] AIFA Agenzia Italiana del Farmaco Riassunto delle Caratteristiche del Prodotto-Duloxetina. https://medicinali.aifa.gov.it/it/#/it/dettaglio/0000057680.

[57] AIFA Agenzia Italiana del Farmaco Riassunto delle Caratteristiche del Prodotto-Amitriptilina. https://medicinali.aifa.gov.it/it/#/it/dettaglio/0000005701.

[58] AIFA Agenzia Italiana del Farmaco Riassunto delle Caratteristiche del Prodotto-Pregabalin. https://medicinali.aifa.gov.it/it/#/it/dettaglio/0000054459.

[59] AIFA Agenzia Italiana del Farmaco Riassunto delle Caratteristiche del Prodotto-Nicetile. https://medicinali.aifa.gov.it/it/#/it/dettaglio/0000015935.

[60] Nguena Nguefack H.L., Gabrielle Pagé M., Guénette L., Blais L., Diallo M., Godbout-Parent M., Angarita-Fonseca A., Lacasse A. (2022). Gender Differences in Medication Adverse Effects Experienced by People Living With Chronic Pain. Front. Pain Res..

[61] Khan J.S., Hah J.M., Mackey S.C. (2019). Effects of smoking on patients with chronic pain: A propensity-weighted analysis on the Collaborative Health Outcomes Information Registry. Pain.

